# Astrocyte Aquaporin Dynamics in Health and Disease

**DOI:** 10.3390/ijms17071121

**Published:** 2016-07-13

**Authors:** Maja Potokar, Jernej Jorgačevski, Robert Zorec

**Affiliations:** 1 Laboratory of Neuroendocrinology—Molecular Cell Physiology, Institute of Pathophysiology, Faculty of Medicine, University of Ljubljana, Zaloška 4, 1000 Ljubljana, Slovenia; maja.potokar@mf.uni-lj.si (M.P.); jernej.jorgacevski@mf.uni-lj.si (J.J.); 2 Celica Biomedical, Tehnološki Park 24, 1000 Ljubljana, Slovenia

**Keywords:** astrocyte, glia, aquaporin (AQP), aquaporin isoforms, orthogonal arrays of particles (OAPs), brain edema

## Abstract

The family of aquaporins (AQPs), membrane water channels, consists of diverse types of proteins that are mainly permeable to water; some are also permeable to small solutes, such as glycerol and urea. They have been identified in a wide range of organisms, from microbes to vertebrates and plants, and are expressed in various tissues. Here, we focus on AQP types and their isoforms in astrocytes, a major glial cell type in the central nervous system (CNS). Astrocytes have anatomical contact with the microvasculature, pia, and neurons. Of the many roles that astrocytes have in the CNS, they are key in maintaining water homeostasis. The processes involved in this regulation have been investigated intensively, in particular regulation of the permeability and expression patterns of different AQP types in astrocytes. Three aquaporin types have been described in astrocytes: aquaporins AQP1 and AQP4 and aquaglyceroporin AQP9. The aim here is to review their isoforms, subcellular localization, permeability regulation, and expression patterns in the CNS. In the human CNS, AQP4 is expressed in normal physiological and pathological conditions, but astrocytic expression of AQP1 and AQP9 is mainly associated with a pathological state.

## 1. Water Homeostasis, Brain Edema, and Astrocytes

Water homeostasis in the central nervous system (CNS) is tightly regulated, as even minute changes in extracellular volume affect ion concentrations and, consequently, neuronal excitability [1]. Dysregulated distribution of water in the neural tissue often occurs in brain tumors, brain abscess, meningitis, stroke, and neurotrauma, when brain edema forms and often worsens the outcome of these disorders [2,3]. As the brain is encapsulated within the cranium, it has very limited space for volume enlargement during brain edema. Therefore, mechanisms for efficient and quick redistribution of water within the brain parenchyma are essential for normal neuronal function. Such mechanisms are still largely unclear and, therefore, the possibilities for medical intervention when brain edema develops are limited. Vasogenic edema, which is amenable to treatment, results mainly from increased permeability of the blood–brain barrier [4], whereas cytotoxic edema results from cellular swelling, mainly caused by failure of energy metabolism [3]. Although cell swelling can be, at least in part, attenuated by adrenergic stimulation [5], currently there appears to be no efficient treatment for this event.

Astrocytes are the only cells in the CNS that undergo rapid changes in volume [6,7,8]. These cells populate the gray and white matter of the CNS and are, arguably, the most heterogeneous (in form and function) type of glia [9,10]. Astrocytes can be broadly defined as primary homeostatic cells of the brain responsible for a wide variety of functions that include, for example, regulation of synaptogenesis, synaptic maturation, neurotransmitter homeostasis, brain microcirculation, brain metabolism, and control over the formation and maintenance of the blood–brain barrier [8,11,12,13,14,15,16,17,18,19,20,21,22,23]. All of these processes depend, to a large extent, on the mechanisms by which astrocytes communicate with the surrounding cells. These include plasma membrane channels, receptors, transporters, and mechanisms that mediate the exchange of molecules by exo- and endocytotic processes [24,25,26,27,28,29,30]. Exo- and endocytotic processes involve vesicles, from which signaling molecules are released in the extracellular space, or membrane channels, such as aquaporins (AQPs), that are integrated in the plasma membrane. Both exo- and endocytotic vesicles are mobile in the cytoplasm [31,32] and their mobility is altered under pathologic conditions [33,34].

Non-neuronal cells, which include astrocytes, outnumber neurons in some areas in the CNS, such as the neocortex [35]. Astrocytic abundance in these areas and their anatomical occupancy of non-overlapping territories between other glial cells, neurons, and endothelial cells of the vasculature [11,13,14,15,36,37], places them ideally to enhance transport of molecules across the brain parenchyma, including the transport of water. In addition to extensive research on astrocytes in several fields, such as ion and pH homeostasis, metabolic support to neurons, and modulation of synaptic strength, their role in the regulation of brain water homeostasis is also a topic of interest. Namely, in astrocytes, specialized membrane proteins have been identified that allow fast transmembrane flux of water [38,39,40].

## 2. Aquaporin Types and Their Isoforms in Astrocytes

**Figure 1:**
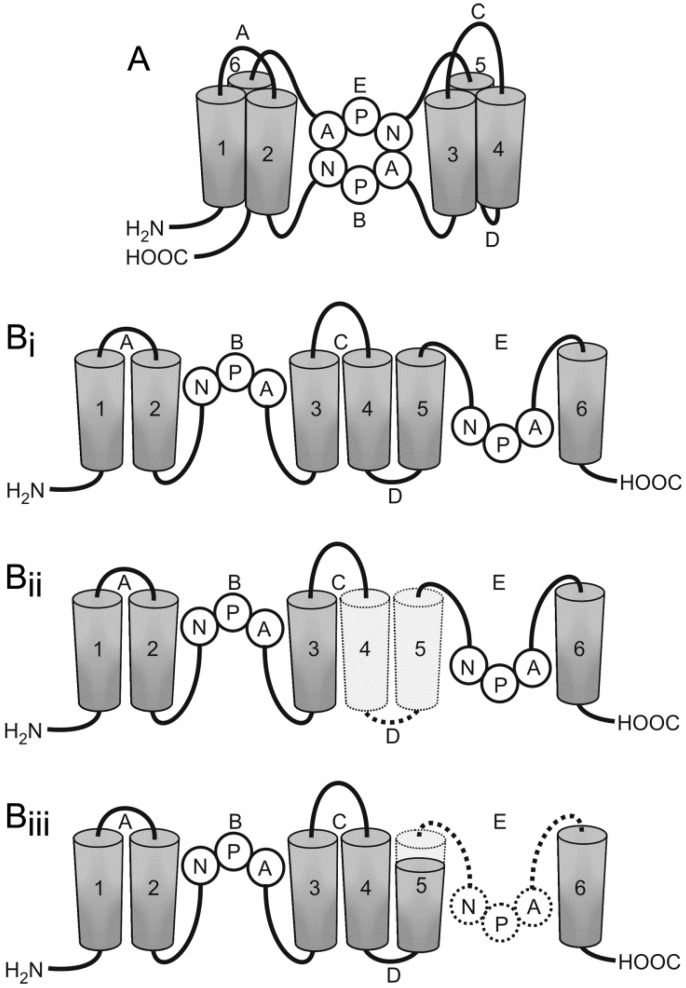
Aquaporin 4 (AQP4) isoforms differ in structure. (**A**) The proposed hourglass model of AQP4. Loops B and E contain NPA motifs (Asn-Pro-Ala), which form an aqueous pore in the membrane bilayer; and (**B**) schematic representations of the AQP4 channel and its isoforms. (**i**) AQP4a, AQP4c, and AQP4e have six bilayer-spanning domains (1–6) and five interconnecting loops (A–E); (**ii**) AQP4b, AQP4d, AQP4f isoforms lack helices 4 and 5 together with their interconnecting loop D; and (**iii**) AQP4-Δ isoform lacks the final part of helix 5 and loop E.

AQPs have been identified in multiple mammalian tissues as well as in invertebrates, plants, and microbes [41]. As their name suggests, APQs are proteins that are specialized in water transport across the plasma membrane of cells, although they are also involved in several other functions [39]. The plasma membrane, per se, is permeable to water molecules, which pass between the intracellular and extracellular space. However, such passive transport is slow, highlighting the need for specialized transmembrane water channels, AQPs, which allow several orders of magnitude higher rates of facilitated transmembrane water flux in the direction of greater osmolality. All AQPs are small hydrophobic proteins, composed of six non-polar bilayer-spanning domains, interconnected by loops A to E (this general description of AQPs structure is evident in the schematic representation of AQP4 depicted in the Figure 1). According to the hourglass model, the conserved loops B and E are essential for the water pore [39,42,43,44]. To date, 13 AQP types have been characterized in mammalian cells; three types, AQP1, AQP4, and AQP9, have been confirmed in the CNS [45]. They differ in their permeability properties (Table 1) and have different distribution patterns. AQP1 and AQP4 are categorized as “pure” aquaporins, water channels primarily only permeable to water, whereas AQP9 is an aquaglyceroporin, permeable to water and to small solutes, such as glycerol and urea [45]. AQP1 was the first AQP discovered in the CNS, identified in the epithelium of the choroid plexus and later also in human astrocytes [46,47]. The most abundant AQP in the mammalian brain is AQP4, which is predominantly localized in astrocytes that are in direct contact with capillaries and pia. AQP4 is not confined solely to astrocytes; it has also been found in subpopulations of ependymal cells [40]. The distribution of AQP9 in the brain is unique; it has been identified in ependymal cells lining the ventricles and in tanycytes of the mediobasal hypothalamus. AQP9 is also present in astrocytes, in endothelial cells of pial vessels, and almost exclusively in catecholaminergic neurons [48,49,50].

The strategic anatomical position of astrocytes with regard to water transport is, therefore, mirrored in the fact that all three CNS AQPs are expressed in these cells. We review these AQP types and their isoforms in respect of their expression patterns, intracellular localization, permeability regulation, and their role in normal physiological and pathological conditions.

### 2.1. Aquaporin 1

#### 2.1.1. Aquaporin 1 (AQP1) in the Central Nervous System (CNS): Expression in Physiological and Pathological Conditions

The AQP1 channel is primarily a water pore to facilitate transmembrane transport of water molecules driven by osmotic gradients [51]. AQP1 was the first AQP to be identified, initially found in erythrocytes and renal tubules [38,52,53,54]. Its expression was also confirmed in other tissues as shown in Table 2. In the CNS, AQP1 was first detected in rat choroid plexus epithelial cells where it is restricted to their apical microvilli [55]. Its distribution in the choroid plexus epithelium (CPE) implied a role in the secretion of cerebrospinal fluid (CSF) into ventricles, which was subsequently confirmed [51,55,56,57,58].

In addition to the CPE, the expression of AQP1 was also confirmed in CNS astrocytes. However, in human astrocytes, its expression is mainly restricted to neuropathologic conditions, e.g., multiple sclerosis (MS), Alzheimer disease (AD), Parkinson disease (PD), and amyotrophic lateral sclerosis [47,62,65]. AQP1 in astrocytes has been detected in astrocytomas in subarachnoid hemorrhage tissue, in Creutzfeldt–Jakob disease (CJD), in bovine and murine spongiform encephalopathy, and in a rat epilepsy model [66,67,68,69,70]. Initially, no AQP1 was found in the healthy human neocortex [66], but it was later reported that a minor population of astrocytes expresses AQP1 in a neurologically normal brain [47].

In human astrocytes, the expression of AQP1 is almost exclusively restricted to pathologic CNS tissue, but it has been detected in a healthy non-human primate brain of the *Macaca fascicularis* monkey [63]. Therefore, in at least some primates, astrocytic AQP1 plays an important role in the regulation of water homeostasis in the CNS in non-pathological brain. This role remains to be further elucidated.

What do we know about the intracellular distribution of AQP1 in astrocytes? In cultured human astrocytes, AQP1 was found co-expressed with AQP4 [47]. The detailed intracellular distribution of AQP1 remains to be thoroughly addressed, but it appears that its localization in cultured astrocytes is not exclusive to the plasma membrane; it is also present in the cytoplasm and likely in the nuclear membrane [47,69]. Its abundant intra-astrocyte distribution in cultured cells may be, in part, the consequence of the in vitro conditions, which differ from those in intact tissue, as was observed also for AQP4 [71,72].

However, variable intracellular distribution in astrocytomas has also been described. In low-grade astrocytomas, AQP1 immunoreactivity was present mostly in the plasma membrane, whereas in high-grade astrocytomas, massively-upregulated AQP1 was also distributed throughout the cytoplasm [70]. In addition to the variable intra-astrocytic distribution of AQP1, the expression of AQP1 also varies among different types of astrocytes, as was shown in intact CNS tissue. High expression of AQP1 was detected in highly-branched fibrillary astrocytes, whereas expression was low in poorly-branched protoplasmic and hypertrophic astrocytes. For example, this pattern was observed in MS and AD brain [47,62]. In both cases, AQP1 expression was observed in highly-branched fibrillary astrocytes positioned around blood vessels and neurons in tissue showing degenerative changes. In the MS brain, such astrocytes were present in chronic active demyelinating lesions and in the AD brain, they were in close proximity to β-amyloid plaques. Reactive astrocytes in pathological brain parenchyma can express AQP1, AQP4, or both, as was demonstrated in the tissue section of the motor cortex in a patient with PD [62]. In addition, in AD brain, increased expression of AQP1 in astrocytes has been observed only in the early stages of AD [65,69]. Therefore, the expression of AQP1 in human pathological CNS astrocytes appears to be spatially and temporally regulated.

The upregulation of AQP1 may have a pivotal role in the maintenance of water homeostasis in the CNS under pathologic conditions; however, detailed mechanisms remain to be elucidated [70].

#### 2.1.2. Water Permeability of AQP1

Most of the data on the regulation of AQP1 water permeability comes from studies on *Xenopus* oocytes; AQP1 regulation in astrocytes remains to be determined. In general, water permeability through AQP1 may be regulated at several levels. One of them is phosphorylation. AQP1 has four potential phosphorylation sites that can be phosphorylated by protein kinase A (PKA), protein kinase C (PKC), and calmodulin-dependent kinase II (CKII). These phosphorylation sites are conserved in humans, rats, and mice [73]. Several contradictory results have been reported regarding the effect of AQP1 phosphorylation on water permeability in *Xenopus* oocytes. Phosphorylation of AQP1 by PKA and PKC was reported to increase water channel permeability in *Xenopus* oocytes [74,75], but others reported that phosphorylation by PKA or PKC did not affect water permeability of aquaporins 1–5 [76]. In addition to kinases, atrial natriuretic peptide (ANP) and arginine vasopressin (AVP) have also been shown to modulate water permeability of AQP1 in transfected *Xenopus* oocytes. AVP has been shown to increase and ANP to decrease membrane water permeability [77].

Water permeability of AQP1-expressing cells may also be related to AQP1 redistribution to the plasma membrane. Such cAMP-dependent redistribution of AQP1 has been demonstrated in *Xenopus* oocytes [74]. Similarly, in rat cholangiocytes, increased insertion of AQP1 in the apical membrane triggered by microtubule-dependent secretin was observed [78]. In addition to short-term regulation, long-term regulation of AQP1 water permeability is also important [73]. Both aspects of regulation in astrocytes are still unclear.

### 2.2. Aquaporin 4

#### 2.2.1. AQP4 in the CNS: Expression in Physiological Conditions

AQP4 was first cloned from rat lung and then identified in different tissues (Table 3) [79,80]. It is predominantly expressed in the brain, where it is also the most abundant water channel [39,40,42]. A subpopulation of ependymal cells lining the ventricles also expresses AQP4, but the principal site of AQP4 expression in the CNS is astrocytes [40,80,81,82]. The highest levels of AQP4 expression were recorded in astrocytes along the subarachnoid space, ventricles, blood vessels, and in areas for osmosensation and regulation of body water balance, including the supraoptic nucleus and subfornical organ [40]. Particularly enriched expression of AQP4 occurs in astrocytes in contact with capillaries and pia, indicating that this AQP4 expression is associated with brain–blood or brain–liquor interfaces [40,63]. In addition, AQP4 is also found on astrocytic processes around synapses. Astrocytes enwrapping distinct types of synapses express different amounts of AQP4, i.e., strong immunoreactivity was found in the glial processes in contact with parallel fiber synapses on Purkinje cell dendritic spines, whereas in other synapses astrocyte processes were modestly labeled [40,83,84].

#### 2.2.2. Intracellular Distribution of AQP4

In general, the intracellular distribution of AQP4 in astrocytes is distinctly polarized, predominantly concentrated in the plasma membrane of glial processes close to or in direct contact with blood vessels, the ependymal layer, and pia [40,85]. However, in distinct osmosensory areas, glial processes show little or no polarization of AQP4 distribution [40]. A similar observation was described for rat and mouse astrocyte cultures, where immunolabeling of AQP4 revealed an intracellular and plasma membrane pattern [71,72]. The strong intracellular pattern observed in cultured murine astrocytes may also be a consequence of the extracellular milieu that is altered in comparison with that in intact tissue. By different experimental manipulation, localization of AQP4 in the plasma membrane significantly increased in stellation-induced rat astrocytes [71] and in astrocytes plated on a basement membrane matrix consisting mainly of laminin [72].

Several AQP4 isoforms are recognized in astrocytes (AQP4a–f; Table 4), but the description of AQP4 intracellular distribution is more complex [42,89,90]. The intracellular localization of individual AQP4 isoforms is well described for the two most studied isoforms, AQP4a (M1) and AQP4c (M23), but it is still poorly investigated for other isoforms. Briefly, to date, seven isoforms of AQP4 water channel have been described; AQP4a–f, which were all detected in astrocytes, and AQP4-Δ [89,91]. The first two AQP4 isoforms to be described in humans, rats, and mice are AQP4a (M1) and AQP4c (M23), which are alternative transcripts from two different initiating methionine sites [42,79,90,92,93]. In rat CNS, the shorter AQP4c (M23; 32 kDa) isoform was shown to be more abundant than the longer AQP4a (M1; 34 kDa) isoform [90,94].

A thorough mapping of the rat *AQP4* gene revealed four additional isoforms besides the two classic isoforms: AQP4a (M1) and AQP4c (M23) [89]. Hence, a new uniform terminology was proposed. M1 and M23 were renamed as AQP4a and AQP4c, respectively, and additional isoforms were named AQP4b, AQP4d, AQP4e (also termed Mz isoform [96]), and AQP4f [89]. The AQP4e-sized (36 kDa) isoform was later confirmed in mice, pigs, and humans [97]. Plasma membrane water-permeable isoforms AQP4a, AQP4c, and AQP4e are basic isoforms, whereas AQP4b, AQP4d, and AQP4f are their alternative splicing variants [89]. The latter three isoforms have so far been shown to be intracellular, and, based on their structure, it is hypothesized that they are unlikely to transport water. All three isoforms lack exon 2, which is why they have only four transmembrane helices; the other three isoforms all have all six (Figure 1). They lack helices 4 and 5 together with their interconnecting loop D, which is one of the stabilizing factors of AQP4. When transfected into *Xenopus* oocytes, they failed to enhance water permeability through their plasma membrane [89]. However, this is not entirely surprising, given their likely intracellular localization.

Different AQP4 isoforms have distinct localization at the subcellular level. AQP4a (M1) and AQP4c (M23) are predominantly localized at the plasma membrane of perivascular endfeet regions of astrocytes. Although, in addition to their strong distribution in the plasma membrane, intracellular localization was also observed in cultured astrocytes [85,90,95]. Localization of AQP4e in the plasma membrane has already been confirmed in rat brain lysates, *Xenopus* oocytes, HeLa cell lines, human malignant glioblastoma cell lines, and in primary cultured rat astrocytes [72,89,96,98]. The abundance of its expression in the plasma membrane of astrocytes appears to be dynamic, affected by the osmolality of the extracellular milieu [72]. In addition to the plasma membrane, internal AQP4e localization has also been observed in HeLa cell lines, as well as cultured rat astrocytes, consistent with the recycling of AQP4 [72,89,99]. Intracellularly, the AQP4e isoform was found in the Golgi apparatus (GA) and in late endosomal degradation compartments [72]. Moreover, it was also observed in highly dynamic vesicles, the mobility of which was impaired in a model of reactive gliosis and at high levels of intracellular concentration of calcium ions. In hypo-osmotic conditions, mimicking cell edema, the mobility of vesicles carrying the AQP4e isoform changed in different intervals after hypo-osmotic stimulation and was inversely correlated with the abundance of AQP4 at the plasma membrane. Decreased mobility overlapped with increased plasma membrane localization [72].

AQP4b, AQP4d, and AQP4f isoforms remained situated intracellularly when expressed in *Xenopus* oocytes and in HeLa cells and all of them were shown to colocalize with GA and showed a broader cytoplasmic distribution [89]. In cultured astrocytes, AQP4d was also detected intracellularly in GA and in late endosomal degradation compartments [72]. To understand the role of the intracellular AQP4 isoforms in astrocytes, especially those newly detected, there is a need to systematically investigate their subcellular localization and function.

In addition to AQP4b, AQP4d, and AQP4f, another intracellular AQP4 isoform was described in skeletal muscle. This is alternatively spliced transcript named AQP4-Δ4 that lacks exon 4. AQP4-Δ4 lacks the final part of transmembrane helix 5 and loop E, which contains the second asparagine-proline-alanine (NPA) motif essential for the formation of the structural domain for water permeation in the aquaporin monomeric channel (Figure 1). NPA motifs form the loops connecting helices 2 and 3, and 5 and 6, on the opposite sides of the membrane bilayer [42,91,100,101]. AQP4-Δ4 shows no water transport properties in HeLa cells and was shown to reside in the ER, although a minor amount of AQP4-Δ4 was detected at the plasma membrane. AQP4-Δ4 was proposed to downregulate the expression and activity of AQP4, which likely originates from a dominant-negative effect caused by heterodimerization between AQP4 and AQP4-Δ4 [91]. Similar to the intracellular AQP4 isoforms, several other aquaporins are localized in intracellular membrane compartments, such as AQP2, AQP6, AQP8, AQP10, and AQP11 [102,103,104,105,106,107].

#### 2.2.3. Orthogonal Arrays of Particles

A unique property of the AQP4 water channel is the formation of higher-order structures in the plasma membrane. First, monomers assemble into tetramers, which then aggregate into larger structures called orthogonal arrays of particles (OAPs) [108]. OAPs have been identified in several mammalian tissues and have been named in line with their appearance (orthogonal lattices) when visualized by freeze-fracture electron microscopy [109]. OAPs of AQP4 have been observed in astrocytes, trachea, sarcolemma, gastric parietal cells, kidney principal cells, ciliary body, and the intestine [80]. In the CNS, extensive OAPs are found in perivascular astrocyte endfeet and in astrocyte processes of the glia limitans beneath the pia [85,94,110]. Here, AQP4 tetramers (4–6 nm in size) are mostly (>90%) present as OAPs [94]. OAPs in astrocytes are formed from at least two AQP4 isoforms, AQP4a (M1) and AQP4c (M23). Although AQP4c (M23) has the intrinsic ability to form OAPs alone through specific intermolecular N terminus interactions, AQP4a (M1) can form OAPs only when expressed together with AQP4c (M23). OAPs assembled from AQP4a and AQP4c together are smaller than OAPs composed of AQP4c alone, showing that these two isoforms have opposing effects on OAP size [94,110,111,112,113].

In addition to AQP4a (M1) and AQP4c (M23), the AQP4e (Mz) isoform has also been proposed to assemble into OAPs, but only together with AQP4c (M23), similar to the AQP4a (M1) isoform [96,98]. There is a possibility that, in the tissue, OAPs contain all three plasma membrane water permeant isoforms, AQP4a (M1), AQP4c (M23), and AQP4e (Mz), as observed in brain lysates and in the transfected U87MG (human glioblastoma-astrocytoma) cell line [96,98]. The properties of AQP4e assembly into OAPs and the function of AQP4e in OAPs remain to be established, as well as the role of OAPs themselves. Several hypotheses have been proposed regarding the role of OAPs, including acceleration of water transport across the astrocyte plasma membrane, adhesive functions, clearance of macromolecules from brain interstitium, and optimizing gas exchange between blood and the brain [108,114,115]. However, none of these roles has been unequivocally confirmed.

#### 2.2.4. AQP4 Water Permeability Regulation in Astrocytes

Knowledge on the water permeability of AQP4 in astrocytes is scarce. In general, it is hypothesized that water permeability through AQP4 channels can be regulated at several levels. In brief, these are phosphorylation of the channel, expression and density of AQP4 channels at the plasma membrane, the rate of AQP4-laden vesicle delivery to the plasma membrane, and aggregation of AQP4 into OAPs.

AQP4 has several potential phosphorylation sites [73], which makes the regulation of water permeability by phosphorylation plausible. However, in cultured rat astrocytes, AQP4 failed to be phosphorylated by PKA and not all of the phosphorylation sites affect water permeability, as was shown in the case of Ser111 [71,116,117]. On the other hand, activation of PKC has been shown to phosphorylate Ser180 of AQP4, which resulted in reduced water permeability and inhibition of cell migration in a glioma cell line [118]. In cultured rat astrocytes, PKC also decreased AQP4 mRNA and protein expression, probably through signal transduction [119].

Another factor that can affect water permeability through AQP4 in astrocytes is the abundance of AQP4 expression at the plasma membrane. Fast changes in the expression of AQP4 in the plasma membrane can be regulated by translocation of the channel to/from the plasma membrane via membrane-bound vesicles of the existing cellular AQP4 pool [72]. However, the possible redistribution of the AQP4 channel to/from or inside the plasma membrane has not been addressed in the tissue, where most of the AQP4 signal is observed at the plasma membrane. Changes in the expression levels of AQP4 in the plasma membrane were observed under several pathological conditions. These changes may in part involve deregulation in vesicle traffic, as in astrocytes from an AD mouse model [120]. Altered expression levels of AQP4 in pathological conditions are reviewed in detail in the following section.

It is also hypothesized that the formation of OAPs in the plasma membrane can affect water permeability of the astrocyte plasma membrane. This could be achieved through differential assembly of OAPs in a post-Golgi phase governed by different expression levels of the isoforms that are also tissue specific [94,115,121,122,123,124]. In addition, other factors regulating short-term OAP assembly/modifications need to be addressed.

#### 2.2.5. Altered Expression of Astrocytic AQP4 in Pathologic Conditions

Under several pathologic conditions, alterations in the subcellular distribution of AQP4 and its expression levels have been reported in astrocytes. The abundance of AQP4 in the plasma membrane has been observed to either decrease or increase, and it may be linked to altered expression of the *AQP4* gene or to complementary proteins. For example, an extensive decrease in the abundance of AQP4 channels in the plasma membrane was detected in the perivascular plasma membrane of astrocytes in the hippocampal area of patients with epilepsy. This decrease was attributed to decreased expression of the AQP4 anchoring protein, even though AQP4 mRNA expression increased in the same reactivated astrocytes [125,126]. Apparent discrepancies have been noted in several reports on AQP4 expression in the plasma membrane of patients with AD. The reasons for the observed decreases or increases in AQP4 in the plasma membrane remain to be clarified; however, they may arise due to samples from different stages of the disease and from different brain regions. Nonetheless, increased expression of AQP4 was observed in patients with AD with or without cerebral amyloid angiopathy (CAA), where extensive AQP4 immunoreactivity was seen around blood vessels in the CSF and brain interfaces [127]. Similarly, increased AQP4 expression was detected in cortical sections of temporal lobes of patients with AD, where AQP4 immunostaining was more intense around larger vessels or capillaries affected by CAA and it varied depending on the severity of CAA. Increased AQP4 expression was detected around senile plaques, where it was increased during early β-amyloid (Aβ) peptide deposits and was downregulated in the later stage of Aβ plaque formation [128]. On the other hand, no differences in the level of AQP4 expression were reported after Western blotting of the frontal cortex samples from patients with AD, although glial fibrillary acidic protein labeling revealed moderate astrogliosis [65,69]. Changes in the expression of AQP4 in the plasma membrane was also screened in a mouse model of AD, where an increased concentration of AQP4 was observed in astrocytic processes in synaptic regions and a decrease in AQP4 abundance in astrocyte endfeet membranes, specifically at sites of perivascular Aβ deposits [129]. Increased expression levels of AQP4 were also reported in the brain of patients with CJD, in particular in the cytoplasm of protoplasmic and fibrillary astrocytes in the cerebral cortex and white matter, respectively [69]. AQP4 immunoreactivity in astrocytes was also more abundant in brain from patients with MS, especially at the periphery of plaques [130].

In addition to neurodegenerative diseases, astrocytic AQP4 expression was upregulated in human tissue in several other pathologic conditions, such as edematous brain tumors and surrounding tissue, after subarachnoid hemorrhage, and in peritumoral tissue and ischemia [66,131,132]. The expression of AQP4 was increased in the astrocytic processes, but in certain examples, polarization on astrocytic endfeet was lost [66].

Taken together, an extensive decrease of AQP4 expression in epilepsy patients likely results in perturbed water and ion homeostasis, leading to an increased propensity for seizures and cognitive decline [129]. Upregulated plasma membrane expression of AQP4 is hypothesized to facilitate the transport of water through blood vessel walls, as well as pial and ependymal surface of the brain and contribute to the development of brain edema [125,126].

### 2.3. Aquaglyceroporin 9

#### 2.3.1. AQP9 in the CNS: Expression in Physiological Conditions

AQP9 is an aquaglyceroporin and is the least studied AQP in astrocytes. Like all aquaporins, it is permeable to water but also to small solutes. When expressed in *Xenopus* oocytes, human AQP9 was found to be permeable to a variety of structurally-unrelated solutes, including polyols (glycerol, mannitol, sorbitol), purines (adenine), pyrimidines (uracil and the chemotherapeutic agent 5-fluorouracil), and urea analogs (thiourea) [133,134,135]. In general, the expression of AQP9 has been identified in different cell types, including astrocytes (Table 5).

In mouse brain astrocytes, expression of AQP9 was observed in the processes bordering the subarachnoid space and ventricles, in the white matter, hippocampus, hypothalamus, and lateral septum [48]. In addition, in the adult rat brain, astrocytes expressing AQP9 were detected in the white matter and gray matter [49]. AQP9 was also detected in the astrocytes of a non-human primate brain [63]. As in rodent species, AQP9 mRNA and protein were detected in other CNS cells [63]. Reports of human expression of AQP9 are scarce and are limited mainly to pathological tissue, such as astrocytomas [136,137].

#### 2.3.2. Intracellular Distribution of AQP9

Several AQP9 isoforms have been identified to date. For example, in astrocytes and in a subpopulation of neurons (dopaminergic neurons in the substantia nigra and ventral tegmental area) isolated from rat brain, two AQP9 isoforms have been identified: ~25 and ~30 kDa isoforms. The ~30 kDa isoform is expressed in the plasma membrane and may correspond to the liver isoform (~32 kDa) or is possibly even a splicing variant. On the other hand, the ~25 kDa isoform (obtained by alternative splicing) is expressed in mitochondria and is highly enriched in the inner mitochondrial membrane of astrocytes. Its major role could be the transport of lactic acid into the mitochondria, which would benefit cells in ischemic conditions [140].

In general, AQP9 immunostaining in astrocytes was observed in their cell bodies, in processes directed toward blood vessels, and in perivascular endfeet; so it differs from AQP4 polarized immunolabeling [49,63]. In contrast, AQP9 forms only tetramers in cellular membranes and not higher-order complexes like AQP4 [98].

#### 2.3.3. The Role of AQP9 in the CNS

Several functions of AQP9 in the CNS have been considered. AQP9 permeability to diverse molecules implies its role in diverse processes, such as water homeostasis and energy metabolism. Among several hypotheses, it was suggested that AQP9 may facilitate clearance of lactate and glycerol from the extracellular space, as is the case in cerebral ischemia [48]. Given that it is localized in the glia limitans and tanycytes, it may also contribute to water flow in CSF between the brain parenchyma and subarachnoid space [49]. Considering that AQP9 facilitates glycerol diffusion, as reported recently, it is probably also involved in energy metabolism in the CNS. Silencing of AQP9 in cultured astrocytes induced a decrease in glycerol uptake and triggered changes in astrocyte energy metabolism by increasing the glucose uptake [141]. Further investigation is needed to elucidate the processes regulated by AQP9 in the CNS in detail.

#### 2.3.4. Permeability Regulation and Expression of AQP9 in Pathological Conditions

As in the case of AQP1 and AQP4, AQP9 can be regulated through phosphorylation, although only a few studies have investigated this. Experiments in cultured rat astrocytes suggest that signal transduction via PKC may decrease plasma membrane expression of AQP9 through PKC activation, and increase expression of AQP9 through the PKA-dependent increase of dbcAMP [116,119].

In addition, rendered plasma membrane expression of AQP9 was reported to be influenced by changes in mRNA expression. For example, an increase in mRNA and protein expression of AQP9 has been measured in human astrocytic tumors (reaching higher values in high-grade tumors) compared with normal brain tissue [136]. Overexpression of AQP9 was also detected in mouse astrocytes in peri-infarct areas after focal transient ischemia [48].

Short- and long-term permeability regulation of AQP9 in astrocytes remains to be elucidated.

Changes in the permeability regulation and expression of AQP9 are hypothesized to play a role in the regulation of postischemia edema and in the clearance of lactate from the damaged tissue, as well as in the malignant progression of brain astrocytic tumors [48,130].

## 3. Conclusions

Three different AQP types have been identified in astrocytes. The most abundant among them is AQP4, which is also the most studied AQP type in the brain. Its expression has been confirmed in human and non-human mammalian brain astrocytes in physiological and pathological conditions. Despite its recognized general role in the regulation of brain water homeostasis, several issues remain to be elucidated, especially in light of newly-described isoforms, their roles, and permeability regulation. In addition to water permeability through the plasma membrane, AQP4 may be implicated in various other roles, such as cell adhesion, gas exchange, and possibly other roles, especially if we consider the growing body of data on different isoforms. Although AQP1 and AQP9 are expressed in physiologically normal tissue of non-human mammalian brains, it appears that, in human astrocytes, they are expressed mainly in pathological conditions. AQP9 is the least studied of all AQP types in astrocytes; its localization in human brain has been unambiguously confirmed only in astrocytomas so far. Variations in astrocytic expression patterns of different AQP types and their permeability properties suggest that they have different roles in maintaining brain homeostasis that need to be elucidated from the perspective of efficient pharmacological manipulation.

## Figures and Tables

**Table 1:** Aquaporin (AQP) types in astrocytes.

AQP Type	AQP Isoforms	Permeability to Water	Permeability to Small Solutes (i.e., Glycerol, Urea, Monocarboxylates)	Ability to Form OAPs in the Plasma Membrane
AQP1	AQP1	Yes	No	No
AQP4	AQP4a (M1)	Yes	No	Only together with AQP4c
	AQP4c (M23)	Yes	No	Yes
	AQP4e	Yes	No	Only together with AQP4c
	AQP4b	?	No	N/A
	AQP4d	?	No	N/A
	AQP4f	?	No	N/A
AQP9	AQP9 ~32 kDa (is AQP9 ~30 kDa its splicing isoform?)	Yes	Yes	No
	AQP9 ~25 kDa	Yes	Yes	No

?, not yet definitely determined; N/A, not applicable; OAPs, orthogonal arrays of particles.

**Table 2:** The expression of AQP1 in different cells.

Cell Type	Reference
Erythrocytes	[53]
Renal epithelial cells	[53]
Endothelial cells (except central nervous system (CNS))	[55]
Epithelial cells of the choroid plexus	[55,59]
Epithelial cells of the iris, ciliary body, lens, trachea, kidney, colonic crypt, sweat glands, pancreatic acini, gallbladder epithelium, placental syncytial trophoblast cells	[55,59]
Sensory nerve fibers in the dorsal horn of the spinal cord and the trigeminal sensory ganglia	[60,61]
Reactive astrocytes (human CNS) in Alzheimer disease, Creutzfeldt–Jakob disease, multiple sclerosis, and in ischemic lesions	[47,62]
Astrocytes (non-human primate CNS, a subpopulation of white matter astrocytes in *Macaca fascicularis*)	[63]
Schwann cells (*Macaca fascicularis* CNS)	[63]
Trigeminal nerve fibers (*Macaca fascicularis* CNS)	[63]
Neurons on the surface of the pial blood vessels (*Macaca fascicularis* CNS)	[63]
Vascular smooth muscle cells	[64]

**Table 3:** The expression of AQP4 in different cells.

Cell Type	Reference
Astrocytes (brain and spinal cord)	[42,79,80,85]
A subpopulation of brain ependymal cells	[40,80,81,85]
Retina, iris, ciliary body	[79,81,82]
Lung epithelial cells	[79,81]
Renal basolateral plasma membrane of collecting duct principal cells, renal papillary vasa recta	[79,81]
Colon (villus) epithelial cells	[81]
Stomach parietal cells	[80]
Excretory tubules of salivary and lacrimal glands	[80]
Auditory epithelium of the organ of Corti	[86,87]
Skeletal muscle; the sarcolemma of fast-twitch fibers	[80,88]

**Table 4:** AQP4 isoforms.

AQP4 Isoforms	Cell Type	Intracellular Localization	Reference
AQP4a (M1)	Astrocytes	PM	[85,90,95]
AQP4c (M23)	Astrocytes, skeletal muscle, kidney	PM	[85,90,95]
AQP4e (Mz)	Astrocytes (rat), organ of Corti (rat)	PM, intracellular vesicles, GA, EC	[72,89,96]
AQP4b	Astrocytes (rat)	GA	[89]
AQP4d	Astrocytes (rat)	GA, EC	[72,89]
AQP4f	Astrocytes (rat)	GA	[89]
AQP4-Δ	Skeletal muscle	ER, faintly in the PM	[91]

PM, plasma membrane; ER, endoplasmic reticulum; EC, late endosomal compartments; GA, Golgi apparatus.

**Table 5:** The expression of AQP9 in different cell types.

Cell Type	Reference
Spinal cord and brain: Astrocytes, ependymal cells lining the ventricles and tanycytes, catecholaminergic neurons, endothelial cells of pial vessels, Bergmann glia	[49,50,138]
Hepatocytes	[50,139]
Testis Leydig cells	[50]
Epididymis stereocilia	[50]
Spleen leukocytes	[50]

## Abbreviations

AQP	aquaporin
OAP	orthogonal array of particles
CNS	central nervous system
CPE	choroid plexus epithelium
CSF	cerebrospinal fluid
MS	multiple sclerosis
AD	Alzheimer disease
PD	Parkinson disease
CJD	Creutzfeldt–Jakob disease
CAA	cerebral amyloid angiopathy
PKA	protein kinase A
PKC	protein kinase C
